# *MMP2* rs243866 and rs2285053 Polymorphisms and Alzheimer’s Disease Risk in Slovak Caucasian Population

**DOI:** 10.3390/life13040882

**Published:** 2023-03-26

**Authors:** Agata Ocenasova, Ivana Shawkatova, Juraj Javor, Zuzana Parnicka, Gabriel Minarik, Maria Kralova, Iliana Kiralyova, Iveta Mikolaskova, Vladimira Durmanova

**Affiliations:** 1 Institute of Immunology, Faculty of Medicine, Comenius University in Bratislava, 811 08 Bratislava, Slovakia; 2 Medirex Group Academy, Ltd., 820 16 Bratislava, Slovakia; 3 Clinic of Psychiatry, Faculty of Medicine, University Hospital, Comenius University in Bratislava, 813 69 Bratislava, Slovakia; 4 Care Centre Centrum Memory, 851 03 Bratislava, Slovakia

**Keywords:** matrix metalloproteinase 2, Alzheimer’s disease, polymorphism, genotyping

## Abstract

Alzheimer’s disease (AD) is an age-related neurodegenerative disorder characterised by progressive loss of memory. In the AD brain, matrix metalloproteinases (MMPs) are involved in the disruption of the blood-brain barrier resulting in a neuroinflammatory response. The objective of our investigation was to assess the association of *MMP2* rs243866 and rs2285053 polymorphisms with susceptibility to AD, to assess the interaction of *MMP2* variants with *APOE* ε4 risk allele, and to evaluate their influence on the age at disease onset and MoCA score. A total of 215 late-onset AD patients and 373 control subjects from Slovakia were genotyped for *MMP2* rs243866 and rs2285053 polymorphisms. The *MMP2* association with AD risk and clinical parameters was evaluated by logistic and linear regression analyses. No statistically significant differences in either *MMP2* rs243866 and rs2285053 allele or genotype frequencies between AD patients and the control group have been observed (*p* > 0.05). However, the correlation with clinical findings revealed a higher age at disease onset in *MMP2* rs243866 GG carriers in the dominant model as compared to other *MMP2* genotype carriers (*p* = 0.024). Our results suggest that *MMP2* rs243866 promoter polymorphism may have an impact on the age at AD onset in the patients.

## 1. Introduction

Alzheimer’s disease (AD) is a degenerative brain disorder that leads to memory loss, behavioral changes, and a decline in brain matter [1]. There are two forms of Alzheimer’s disease: early-onset Alzheimer’s, which affects people under 65, and late-onset Alzheimer’s (LOAD), which is the more common form and accounts for over 90% of cases, affecting those over 65 years of age [2]. The brain of Alzheimer’s patients displays histopathological features such as senile plaques made of amyloid β peptide (Aβ) and neurofibrillary tangles (NFTs) composed of abnormally phosphorylated tau proteins [3,4]. The underlying cause of Alzheimer’s disease is still unknown. However, its progression may be linked to neuroinflammation caused, among others, by matrix metalloproteinases (MMPs) [5]. MMPs belong to the family of zinc-dependent endopeptidases that participate in the remodelling of the extracellular matrix in various physiological and pathological processes [6]. In the brain, MMPs are involved in neurogenesis, axonal growth, angiogenesis, tissue repair after injury, and inflammation [7]. They have been implicated in various brain disorders such as multiple sclerosis, amyotrophic lateral sclerosis, Parkinson’s disease, and Alzheimer’s disease [8]. MMPs contribute to the progression of neurodegenerative diseases by regulating processes such as neuroinflammation, microglial activation, degradation of amyloid peptides, disruption of the blood-brain barrier (BBB), apoptosis of dopaminergic cells, and modulation of α-synuclein [5,9].

Among the MMP family, matrix metalloproteinase 2 (MMP-2), also known as gelatinase A, is currently classified as one of the possible candidates to participate in the pathogenesis of AD. MMP-2 is a 72 kDa protein encoded by the MMP2 gene located on chromosome 16q21 [7]. It is produced by endothelial cells, fibroblasts, osteoblasts, myoblasts cells, and other connective tissue cells. MMP-2 is known for its capacity to degrade a wide range of substrates such as elastin, endothelin, fibroblast growth factor, aggrecans, plasminogen, TGF-β, and others. Moreover, the enzyme is capable of cleaving type IV collagen—an important constituent of the lamina densa of the basement membrane. The MMP-2 synthesis can be activated by a range of pathological stimuli, such as tumour invasion and inflammation [9]. In the brain, MMP-2 plays an important role in neuronal plasticity, neurite outgrowth, and central nervous system development [10].

Increased expression of MMP-2 has been observed in astrocytes surrounding Aβ plaques and brain endothelial cells in Alzheimer’s disease patients and a decrease in MMP-2 plasma levels compared to healthy individuals [11,12,13,14]. In a 5xFAD mouse model, MMP-2 upregulation was shown in areas enriched with Aβ during the early stages of Alzheimer’s disease and continued through the progression of the disease [15]. Additionally, MMP-2 accumulation has been found near NFTs in the early stages of AD, and it has been demonstrated that P-tau stimulates MMP-2 expression [16]. MMP-2 was shown to break down Aβ peptides into nontoxic fragments, suggesting its protective role in AD pathology [11,17,18,19]. MMP-2 also has the capacity to degrade recombinant tau in vitro but is not able to process its hyper-phosphorylated forms present in NFTs [16]. However, increased MMP-2 expression induced by toxic Aβ1-42 oligomers was associated with the disruption of BBB, causing neuroinflammation, synaptic loss, and Alzheimer’s disease progression [18,20,21,22,23].

Studies have found a positive correlation between MMP-2 plasma activity and the Mini-Mental Status Examination (MMSE) score in Alzheimer’s disease patients [12]. Additionally, AD patients with white matter lesions showed significantly higher plasma levels of MMP-2 compared to those without white matter lesions [24]. According to previous studies, MMP-2 has been proposed as one of the CSF biomarkers related to the detection of AD patients [25,26].

Variations in MMP expression may be associated with common polymorphisms in the promoter regions of the corresponding genes. *MMP2* rs243866 (−1575 G>A) and rs2285053 (−735 C>T) are two single nucleotide polymorphisms in the promoter region; they are linked to alterations in MMP-2 expression levels. The −1575 G>A polymorphism is located close to a 5′ half-palindromic potential estrogen receptor binding site and the -1575A allele has been shown to decrease transcriptional activity significantly [27]. A C to T substitution at position −735 has been associated with decreased expression of the *MMP2* gene as well [28,29].

Genetic impact of *MMP2* rs243866 (−1575 G>A) and rs2285053 (−735 C>T) polymorphisms on AD risk have not been analysed until now. However, one study presented a significant association of the rs2285053-T allele with susceptibility to develop HIV-associated neurocognitive disorders [30]. The first objective of the current investigation was to assess the association of *MMP2* rs243866 (−1575 G>A) and rs2285053 (−735 C>T) polymorphisms with disease susceptibility in a group of Slovak patients with late-onset Alzheimer’s disease by a case-control study. Second, we aimed to establish the interaction of these *MMP2* variants with *APOE* ε4 as the known AD risk allele. Moreover, we aimed to evaluate the influence of studied *MMP2* polymorphisms on the age of onset and on the level of cognitive impairment expressed as the total score of Montreal cognitive assessment (MoCA).

## 2. Materials and Methods

### 2.1. Study Subjects

The study included 215 patients with late-onset AD (67 men and 148 women, mean age: 79.45 ± 5.85 years). The diagnosis was assessed by the participating physicians according to the National Institute of Neurological and Communicative Disorders and Stroke and the Alzheimer’s Disease and Related Disorders Association (NINCDS-ADRDA) diagnostic criteria [1]. All AD study subjects were recruited randomly via several psychiatric and neurologic clinics throughout the Slovak Republic. The mean age at disease onset was 77.21 ± 6.16 years. The control group comprised 373 unrelated individuals (147 men and 226 women with a mean age of 76.70 ± 7.73 years) without any personal or family history of AD or other psychiatric disorders. All participants were Caucasians of Slovak descent. As a tool to assess cognitive function, the Montreal Cognitive Assessment (MoCA) was selected [31]. The recommended cut-off score of ≥26 out of a maximum of 30 was applied for normal cognitive performance. *APOE* ε4 allele, as a known genetic risk determinant for AD, was examined in both study cohorts and included as a stratification factor in further analyses. The study groups’ fundamental characteristics are given in Table 1. The study protocol was approved by the Independent Ethical Committee of the Bratislava Municipality under the No. 05440/2021/HF. The study was conducted in accordance with the Declaration of Helsinki and with the International Ethical Guidelines. Informed consent was obtained from all participants prior to their enrollment in the study.

### 2.2. Genotyping

DNA of AD patients and controls was extracted from whole blood samples treated by EDTA using a modified phenol-chloroform procedure [32]. *APOE* genotyping was performed by Sanger sequencing of the fourth exon, as described previously, using the rs429358 (C>T) and rs7412 (T>C) polymorphisms as markers for ε4 allele, respectively [33].

The *MMP2* rs2285053 (C or T allele at position −735) was examined by PCR followed by restriction fragment length polymorphism analysis (RFLP). Forward and reverse primer sequences, PCR settings, and HinfI restriction enzyme (Thermo Fisher Scientific, Waltham, MA, USA) were used as reported by Gannoun et al. [34]. The PCR reaction revealed a 391 bp long product. After cleavage, either a whole 391 bp amplicon (allele C) or two fragments of 338 bp and 53 bp (allele T) were generated. The *MMP2* rs243866 (G or A allele at position −1575) was determined by the PCR-RFLP method as reported by Kiani et al. [35]. A 201 bp PCR fragment was amplified and afterwards digested with the restriction enzyme PagI (Thermo Fisher Scientific, Waltham, MA, USA). After cleavage, either an intact 201 bp PCR fragment bearing G allele or two fragments of 112 bp and 89 bp bearing A variant were generated.

### 2.3. Statistical Analysis

Categorical variables (sex, APOE ε4 presence) were compared between the study groups by the χ^2^ test, whereas differences in continuous variables (age, age of onset, MoCA score) were assessed by the Welch’s corrected *t*-test (for normally distributed data) or Mann–Whitney test (for nonparametric data). The distribution of individual alleles and genotypes was determined by direct counting. *MMP2* genotypes were checked for their departure from the Hardy–Weinberg equilibrium by the χ^2^ goodness-of-fit test. Statistical differences in allele distribution between AD cases and controls were evaluated using the Pearson chi-squared statistic (GraphPad Software, version 3.10, Inc., San Diego, CA, USA). The multivariate logistic regression analysis adjusted for age, sex, and *APOE* ε4 carriage status was employed to examine the association between *MMP2* variants and AD risk under the codominant, dominant, recessive, over-dominant, and log-additive inheritance models. For this purpose, SNPstats web software available at http://bioinfo.iconcologia.net/SNPstats (accessed on accessed on 14 September 2022) was used [36]. The *p*-values and odds ratios (OR) with 95% confidence intervals (CI) were determined as a relative measure of the effects of alleles and genotypes. Regression analysis and synergy factor (SF) measurement were also completed to assess the significance and size of interaction between *MMP2* rs243866 A, *MMP2* rs2285053 T, and the *APOE* ε4 alleles, as previously described [37]. The correlation between *MMP2* rs243866 and rs2285053 gene promoter polymorphisms and clinical findings as age at onset and MoCA score was evaluated by the linear regression analysis. The *p-*value lower than 0.05 was considered statistically significant.

## 3. Results

### 3.1. Characteristics of the Study Groups

Table 1 shows the comparison of characteristics (demographic and clinical) between AD cases and controls. A total of 215 late-onset AD patients and 373 unrelated controls were enrolled in the research. The difference between the AD group and controls in relation to gender was not statistically significant (*p* = 0.05). Women had a higher prevalence in both AD patients (68.83%) and controls (60.59%). The mean age at the time of obtaining the blood sample and performing the MoCA test was significantly higher in the AD group than in controls (79.45 ± 5.85 vs. 76.70 ± 7.73 years; *p* < 0.0001). In relation to this, the age at examination was included as a possible modifying factor in the statistical analysis. As awaited, the cognitive impairment screening revealed that the MoCA score was lower in AD patients compared to controls (15.47 ± 5.95 vs. 27.61 ± 1.38; *p* < 0.0001). The carriers of the *APOE* ε4 risk allele were significantly overrepresented in the AD cases compared to controls (42.33% vs. 18.50%, *p* < 0.0001). The age at disease onset was obtained in AD patients (mean age 77.21 ± 6.16 years).

### 3.2. Association of MMP2 rs243866 and rs2285053 Polymorphisms in the Promoter Region with AD Risk

Table 2 and Table 3 summarise allele and genotype frequencies of *MMP2* rs243866 (−1575 G>A) and rs2285053 (−735 C>T) observed in AD cases and the control group. Genotype frequencies of both polymorphisms fit the Hardy–Weinberg equilibrium in controls (χ^2^ = 0.50, *p* = 0.48 for *MMP2* rs243866; χ^2^ = 0.91, *p* = 0.34 for *MMP2* rs2285053). However, there was a significant departure of both *MMP2* polymorphisms from HWE in AD patients (χ^2^ = 7.04, *p* = 0.008 for *MMP2* rs243866; χ^2^ = 3.89, *p* = 0.049 for *MMP2* rs2285053). Logistic regression analysis of the SNP variants at −1575 G>A and at −735 C>T in the MMP2 gene adjusted for age, sex, and *APOE* ε4 carrier status revealed no statistically significant differences in allele frequencies (*p* = 0.53, OR = 1.09 for *MMP2* rs243866; *p* = 0.35, OR = 1.18 for *MMP2* rs2285053) and also in genotype frequencies (*p* > 0.05, OR = 0.86–1.57 for *MMP2* rs243866; *p* > 0.05, OR = 0.28–1.26 for *MMP2* rs2285053) between the two studied groups. However, we found a higher OR for the rs243866 A allele in the co-dominant and recessive model in the AD patients when compared to the control group (1.52 and 1.57).

The distribution of the *MMP2* rs243866-rs2285053 haplotypes in both the AD group and the control group was also evaluated. Out of the four possible *MMP2* haplotypes, three haplotypes have been observed in both the AD group and the control group. The most frequent was the G–C haplotype (58.60%; 62.20%), followed by the A–C (26.74%; 25.07%) and the G-T haplotype (14.65%; 12.73%). No statistically significant differences in the *MMP2* haplotype frequencies between AD patients and the controls were observed (*p* > 0.05, Table 4). The *MMP2* rs243866 and rs2285053 variants were in strong linkage disequilibrium (D′ = 0.999).

Stratification of both study groups according to their *APOE* ε4 carriage status as a known risk determinant for AD development was also provided. No statistically significant differences in the distribution of *MMP2* −1575 G>A and *MMP2* −735 C>T genotypes between AD patients and the control subjects in *APOE* ε4-positive and *APOE* ε4-negative groups were shown (Tables S1 and S2).

In order to determine the interaction between the *APOE* ε4 allele and the *MMP2* rs243866 and rs2285053 polymorphisms, we evaluated the combined gene effects of both *MMP2* gene variants and the *APOE* ε4 alleles on LOAD risk (Table 5 and Table 6). There was a significantly increased frequency of carriers of the *APOE* ε4 allele but not the *MMP2* rs243866 A allele (*p* < 0.0001), followed by carriers of both *MMP2* rs243866 A and *APOE* ε4 alleles (*p* < 0.0001) in the AD group as compared to the controls. The observed combined effect size of the *MMP2* rs243866 A and *APOE* ε4 alleles was lower (OR = 3.68) than the predicted joint OR expecting independent effects of both alleles (OR = 4.83), suggesting an antagonistic interaction between them (SF = 0.76). This difference was not significant (*p* = 0.48, Table 5). Similarly, no statistically significant interaction was detected between *MMP2* rs2285053 T and the *APOE* ε4 alleles on LOAD risk (*p* = 0.83, SF = 0.91, Table 6).

### 3.3. MMP2 rs243866 and rs2285053 Genotypes and Main Clinical Features in AD Patients

The association between *MMP2* rs243866 (−1575 G>A) and *MMP2* rs2285053 (−735 C>T) polymorphisms and clinical variables such as MoCA score and age at disease onset were also investigated. The linear regression analysis adjusted for sex and *APOE* ε4 revealed that *MMP2* rs243866 GG carriers in the dominant model had higher age at disease onset when compared to A allele carriers (GG vs. GA + AA: 77.97 ± 6.01 vs. 76.19 ± 6.27, *p* = 0.024, Table 7). On the other hand, in *MMP2* rs2285053 (−735 C>T) genotypes and age at disease onset no significant association was found (*p* > 0.05, Table 8). The analysis of the correlation of the MoCA score with both *MMP2* genotypes did not show any significant differences (*p* > 0.05, Table 7 and Table 8).

## 4. Discussion

Matrix metalloproteinases (MMPs), known as matrixins, are calcium-dependent zinc-containing endopeptidases that are involved in the degradation of the extracellular matrix components and basement membrane compounds [7]. Disruption of blood-brain barriers by MMPs leading to neuroinflammation makes a major contribution to the progression of a vast number of neurological diseases, including Alzheimer’s disease [5]. On the other hand, many MMPs are involved in the degradation of toxic Aβ peptides, revealing a protective role of MMPs in AD development [11,17,18,19]. Among MMP family members, MMP-2, MMP-3, and MMP-9 are the most abundant in the brain [9]. The role of MMP-2 in the development of AD is of particular interest. There was an increase in MMP-2 expression in astrocytes surrounding amyloid plaques, NFTs, and brain endothelial cells in AD cases [11,12,13,16]. On the contrary, the decrease in MMP-2 plasma level in AD subjects compared to healthy controls was reported [14]. Plasma levels of MMP-2 were significantly increased in AD patients with white matter lesions compared with the patients without white matter lesions [24]. A positive correlation between MMP-2 plasma activity and the MMSE score was also reported [12].

Single nucleotide polymorphism, when found in regulatory regions of the gene, can alter gene expression or its function; therefore, the goal of the study was to assess the association of *MMP2* rs243866 and rs2285053 polymorphisms with AD susceptibility and clinical parameters in the Slovak Caucasian population. The *MMP2* rs243866 at position −1575 (G>A) and *MMP2* rs2285053 at position −735 (C>T) in the promoter sequence have been related to the variations in MMP-2 expression levels. The −1575A allele has been shown to reduce transcription activity [27]. Similarly, the C to T substitution at position −735 is associated with a decreased expression of the *MMP2* gene [28,29]. According to literature sources, the possible influence of *MMP2* rs2285053 (−735 C>T) and rs243866 (−1575 G>A) polymorphisms on AD development has not been analysed until now.

Our study revealed no significant association of the *MMP2* rs2285053 variant at position −735 (C>T) with AD risk and no effect on the age of AD onset and the degree of cognitive dysfunction monitored by the MoCA score. Regarding neurodegenerative disorders, only one study reported that individuals with the *MMP2* −735CT genotype and −735T allele were at higher risk of developing HIV-associated neurocognitive disorders (HAND) [30]. As reported in other research studies, the −735C allele as a high producer was associated with MS risk in female patients [38], in patients with ischemic stroke in a Chinese population [39], and with breast cancer risk [40]. On the other hand, the −735T allele as a low producer was linked with a higher risk of recurrent spontaneous abortion [41], and preeclampsia [42], and with a lower risk in lung cancer patients [43].

Concerning the *MMP2* rs243866 (−1575 G>A), no association with AD risk was found in our study. We also performed the analysis of the association of *MMP2* rs243866 with age at disease onset and MoCA score and found a significant association of the *MMP2* −1575 GG genotype with higher age at disease onset in crude analysis and adjusted models. As *MMP2* −1575G allele is associated with a higher level of protein expression, it can be hypothesized that the GG genotype might have a protective effect on AD development. Concerning other research studies, the −1575G allele as a high producer was associated with a higher risk of colorectal cancer [44] and endometriosis [45]. On the other hand, the −1575A allele as a low producer has a significantly increased risk of metabolic syndrome [46], myocardial infarction [47], and prostate cancer patients with DM and smoking habits [35].

The *APOE* ε4 allele is the most significant genetic risk factor for the development of late-onset AD [48,49,50]. Therefore, the association between *MMP2* rs243866 (−1575 G>A) and *MMP2* rs2285053 (−735 C>T) and LOAD risk in subjects stratified by *APOE* ε4 carrier status were also performed. Analyses of *APOE* ε4-positive and *APOE* ε4-negative cohorts did not reveal statistically significant differences in the distribution of *MMP2* variants between cases of AD and the control subjects.

Furthermore, we also evaluated the statistical interaction between the *MMP2* rs243866-A, MMP2 rs2285053-T alleles, and the *APOE* ε4 allele on LOAD risk and calculated the synergy factor value. Although we observed a tendency to antagonistic interaction between *MMP2* rs243866-A, rs2285053-T, and *APOE* ε4 alleles in AD risk, this effect was not statistically significant. It is not clear whether there is a relationship between *MMP2* rs243866-A and rs2285053-T and *APOE* ε4 alleles leading to the biological events in AD subjects. APOE is the major cholesterol transporter within the brain and appears to have a role in the induction of AD by multiple mechanisms. It was shown that APOE in *APOE* ε4 carriers accelerates blood–brain barrier breakdown and degeneration of brain capillary pericytes through activation of the cyclophilin A-matrix metalloproteinase-9 pathway, leading to cognitive decline [51]. *APOE* ε4 isoform has been also associated with impaired binding and clearance of Aβ across BBB leading to deposition of amyloid plaques in the brain [52,53]. It was found that Aβ increases IL-1β levels which leads to increased MMP-2 production in the astrocytes [18]. Another study reported that binding of Aβ to the RAGE receptor increased MMP-2 expression in brain endothelial cells [13]. It can be hypothesized that the synergic effect of both *MMP2* and *APOE* ε4 appears to enhance AD-relating neurodegeneration.

We can see a few limitations of the ongoing study. First, the relatively small size of both groups may reduce the probability of detecting associations between *MMP2* variants in the promoter sequences and the risk of late-onset Alzheimer’s disease. A larger number of LOAD cases would be desirable for a replication of the study. However, our present-day results can be considered as useful for sample size planning in future investigations on this topic. Second, the estimation of age at onset may be influenced by various factors such as recognition of the first clinical symptoms as experienced by patients themselves. Interestingly, while the GG genotype of rs243866 seems to be associated with the later disease onset in our study, there were no significant differences in the MoCA score between the three rs243866 genotypes. This could lead to the conclusion that the rate of cognitive decline might be faster in the GG homozygotes. However, relatively low numbers of AA homozygotes among AD patients might have biased our results. Moreover, the MoCA test employed as a single measurement of cognitive functions was not performed in our AD patients at exactly the same time point after the onset of the disease, which likely had an impact on the MoCA score. Therefore, we have subsequently corrected the genotype-phenotype analysis also for disease duration; however, various other factors potentially influencing the cognitive performance in AD patients could have been involved.

As MMP-2 seems to be involved in AD pathogenesis, their utility as therapeutic target has been also examined. One possibility relies on promoting MMP activities resulting in Aβ degradation. A study on the Tg-APP/PS1 mouse AD model reported that hyperoxygenation increases the production of MMP-2, MMP-9, and tPA, leading to reduction of Aβ deposition in the brain and rescued cognitive impairment [54]. Another therapeutic approach could rely on the inhibition of MMP2 expression. Minocycline and GM6001 belong to MMPs inhibitors, which reportedly reduce upregulated MMP-2 and MMP-9, and thus prevent the degradation of blood-brain and blood-CSF barriers and inflammation. Moreover, these inhibitors were able to reduce oxidative stress associated with cerebral amyloid angiopathy in mouse model of AD [21,55,56]. A dual inhibitor of MMP-2 and MMP-9, known as SB-3CT, reduced brain lesion volumes and prevented neuronal loss and dendritic degeneration in an experimental mouse model of traumatic brain injury [57]. Similarly, SB-3CT was shown to improve long-term neurobehavioral outcomes and hippocampus-associated spatial learning and memory after traumatic brain injury in rats [58]. Overall, as MMP-2 plays an important role in neurogenesis, angiogenesis, and neuronal plasticity in the CNS, its modulation in AD patients requires further investigation.

## 5. Conclusions

Our study analysed the role of *MMP2* rs243866 (−1575 G>A) and rs2285053 (−735 C>T) polymorphisms in genetic susceptibility to AD and their influence on clinical parameters such as age at onset and MoCA score. The results didn’t reveal any genetic association of *MMP2* rs243866 and rs2285053 with the risk of AD; however, the correlation of the *MMP2* rs243866 GG genotype with higher age at onset has been observed. Thus, *MMP2* rs243866 variant seems to be one of the possible determinants of age of Alzheimer’s disease onset.

## Figures and Tables

**Table 1:** Demographic and clinical characteristics of Alzheimer’s disease patients and controls.

Parameter	Controls (*n* = 373)	LOAD Total (*n* = 215)	*p*-Value	LOAD Females	LOAD Males	*p*-Value
				(*n* = 148)	(*n* = 67)	
Age (years)	76.70 ± 7.73	79.45 ± 5.85	**<0.0001**	79.73 ± 5.71	78.82 ± 6.16	0.31
Age of onset (years)	-	77.21 ± 6.16	-	77.32 ± 6.16	76.97 ± 6.21	0.71
Sex (females/males)	226/147	148/67	0.05	-	-	-
MoCA score	27.61 ± 1.38	15.47 ± 5.95	**<0.0001**	15.58 ± 5.95	15.27 ± 6.01	0.72
*APOE ε4* positivity	69 (18.50%)	91 (42.33%)	**<0.0001**	62 (41.89%)	29 (43.28%)	0.85

Data are shown as the mean and standard deviation or as number (%). Significant *p*-values are shown in bold. LOAD: late-onset Alzheimer’s disease; MoCA: Montreal Cognitive Assessment.

**Table 2:** Association between *MMP2* rs243866 (−1575 G/A) SNP and LOAD in the whole population.

Allele/ Genotype	LOAD (*n* = 215)	Controls (*n* = 373)	Genetic Model	Logistic Regression Analysis	
				*p-*Value	OR (95% CI)
			Allele contrast (A vs. G)	0.53	1.09 (0.83–1.43)
G	315 (73.26%)	559 (74.93%)	Codominant (GA vs. GG)	0.61	0.90 (0.61–1.33)
A	115 (26.74%)	187 (25.07%)	Codominant (AA vs. GG)	0.20	1.52 (0.79–2.90)
GG	123 (57.21%)	212 (56.84%)	Dominant (AA + GA vs. GG)	0.99	1.00 (0.70–1.44)
GA	69 (32.09%)	135 (36.19%)	Recessive (AA vs. GA + GG)	0.16	1.57 (0.84–2.96)
AA	23 (10.70%)	26 (6.97%)	Over-dominant (GA vs. GG + AA)	0.42	0.86 (0.59–1.25)
			Log-additive	0.54	1.09 (0.83–1.44)

*p*-values, odds ratio (OR), and 95% confidence intervals (CI) for genotype comparisons were adjusted for *APOE* ε4 carrier status, age, and sex. LOAD: late-onset Alzheimer’s disease.

**Table 3:** Association between *MMP2* rs2285053 (−735 C/T) SNP and LOAD in the whole population.

Allele/ Genotype	LOAD (*n* = 215)	Controls (*n* = 373)	Genetic Model	Logistic Regression Analysis	
				*p-*Value	OR (95% CI)
			Allele contrast (T vs. C)	0.35	1.18 (0.83–1.66)
C	367 (85.35%)	651 (87.27%)	Codominant (CT vs. CC)	0.30	1.24 (0.83–1.87)
T	63 (14.65%)	95 (12.73%)	Codominant (TT vs. CC)	0.26	0.29 (0.03–2.90)
CC	153 (71.16%)	282 (75.60%)	Dominant (TT + CT vs. CC)	0.40	1.19 (0.79–1.78)
CT	61 (28.37%)	87 (23.32%)	Recessive (TT vs. CT + CC)	0.23	0.28 (0.03–2.75)
TT	1 (0.47%)	4 (1.07%)	Over-dominant (CT vs. CC + TT)	0.27	1.26 (0.84–1.89)
			Log-additive	0.57	1.12 (0.76–1.63)

*p*-values, odds ratio (OR), and 95% confidence intervals (CI) for genotype comparisons were ad-justed for *APOE* ε4 carrier status, age, and sex. LOAD: late-onset Alzheimer’s disease.

**Table 4:** Association between *MMP2* rs243866-rs2285053 haplotypes and LOAD in the whole population.

rs243866-rs2285053 Haplotype	LOAD (*n* = 215)	Controls (*n* = 373)	Logistic Regression Analysis	
			*p-*Value	OR (95% CI)
G–C	58.60%	62.20%	reference	
A–C	26.74%	25.07%	0.46	1.11 (0.84–1.47)
G–T	14.65%	12.73%	0.49	1.15 (0.78–1.70)
A–T	0.00%	0.00%	-	-

For each haplotype, estimated frequencies are presented. *p*-values, odds ratio (OR), and 95% confidence intervals (CI) for haplotype comparisons were adjusted for *APOE* ε4 carrier status, age, and sex. LOAD: late-onset Alzheimer’s disease.

**Table 5:** Statistical interaction between *MMP2* rs243866 A and *APOE ε4* alleles.

*MMP2* rs243866 A	*APOE* ε4	LOAD (*n* = 215)	Controls (*n* = 373)	Logistic Regression Analysis		SF (*p*-Value)
				*p*-Value	OR (95% CI)	
−	−	68 (31.63%)	171 (45.84%)	reference		0.76 (0.48)
+	−	56 (26.05%)	133 (35.65%)	0.83	1.06 (0.69–1.62)	
−	+	55 (25.58%)	41 (10.99%)	**<0.0001**	4.56 (2.67–7.79)	
+	+	36 (16.74%)	28 (7.51%)	**<0.0001**	3.68 (2.04–6.63)	

The “−” sign means no copies of the allele, while the “+” sign denotes the presence of at least one copy of the allele. *p*-values, odds ratios (OR), and 95% confidence intervals (CI) were adjusted for age and sex. Synergy factor (SF) was computed as the ratio of the observed OR for both factors combined (3.68) to the predicted OR, assuming independent effects of each factor (1.06 × 4.56 = 4.83). Significant *p*-values are shown in bold. LOAD: late-onset Alzheimer’s disease.

**Table 6:** Statistical interaction between *MMP2* rs2285053 T and *APOE* ε4 alleles.

*MMP2* rs2285053 T	*APOE* ε4	LOAD (*n* = 215)	Controls (*n* = 373)	Logistic Regression Analysis		SF (*p*-Value)
				*p*-Value	OR (95% CI)	
−	−	88 (40.93%)	230 (61.66%)	reference		0.91 (0.83)
+	−	36 (16.74%)	74 (19.84%)	0.43	1.22 (0.75–1.96)	
−	+	65 (30.23%)	52 (13.94%)	**<0.0001**	3.85 (2.43–6.10)	
+	+	26 (12.09%)	17 (4.56%)	**<0.0001**	4.27 (2.18–8.36)	

The “−” sign means no copies of the allele, while the “+” sign denotes the presence of at least one copy of the allele. *p*-values, odds ratios (OR), and 95% confidence intervals (CI) were adjusted for age and sex. Synergy factor (SF) was calculated as the ratio of the observed OR for both factors combined (4.27) to the predicted OR, assuming independent effects of each factor (1.22 × 3.85 = 4.70). Significant *p*-values are shown in bold. LOAD: late-onset Alzheimer’s disease.

**Table 7:** Association of *MMP2* rs243866 (−1575 G/A) SNP with the age of disease onset and MoCA score.

Phenotype	Patient Group	Genotypes			Best Model	*p*-Value
		GG	GA	AA		
AOO	Whole	77.97 ± 6.01	76.13 ± 5.72	76.43 ± 7.90	Dominant	0.024 *
	*APOE* ε4 +	76.57 ± 5.82	74.48 ± 5.07	76.45 ± 4.23	Overdominant	0.12 ^†^
	*APOE* ε4 −	79.11 ± 5.97	77.07 ± 5.91	76.40 ± 10.89	Dominant	0.073 ^†^
MoCA	Whole	16.00 ± 6.17	14.41 ± 5.68	16.35 ± 5.38	Overdominant	0.073 ^‡^
	*APOE* ε4 +	16.46 ± 5.92	14.59 ± 5.56	14.25 ± 4.43	Dominant	0.18 ^$^
	*APOE* ε4 −	15.68 ± 6.36	14.31 ± 5.82	18.22 ± 5.70	Overdominant	0.14 ^$^

Age at onset (AOO) and Montreal Cognitive Assessment (MoCA) are presented as the mean and standard deviation. The “−” sign means the absence of the *APOE* ε4 allele, while the “+” sign denotes the presence of one or two of the *APOE* ε4 allele copies. Linear regression analysis was adjusted for * sex and *APOE* ε4 carrier status; ^†^ sex; ^‡^ age, sex, disease duration, and *ApoE* ε4 carrier status; ^$^ age, sex, and disease duration. Significant *p*-values are shown in bold.

**Table 8:** Association of *MMP2* rs2285053 (−735 C/T) SNP with the age of disease onset and MoCA score.

Phenotype	Patient Group	Genotypes			Best Model	*p*-Value
		CC	CT	TT		
AOO	Whole	76.85 ± 6.29	78.10 ± 5.84	78.00 ± 0.00	Dominant	0.18 *
	*APOE* ε4 +	75.81 ± 5.44	76.32 ± 5.76	78.00 ± 0.00	Dominant	0.67 ^†^
	*APOE* ε4 −	77.62 ± 6.79	79.37 ± 5.64	-	Dominant	0.18 ^†^
MoCA	Whole	15.49 ± 6.08	15.29 ± 5.62	23.00 ± 0.00	Dominant	0.96 ^‡^
	*APOE* ε4 +	15.59 ± 5.60	15.24 ± 5.92	23.00 ± 0.00	Dominant	0.95 ^$^
	*APOE* ε4 −	15.42 ± 6.44	15.31 ± 5.56	-	Dominant	0.95 ^$^

Age at onset (AOO) and Montreal Cognitive Assessment (MoCA) are presented as the mean and standard deviation. The “−” sign means absence of the *APOE* ε4 allele, while the “+” sign denotes the presence of one or two *APOE* ε4 allele copies. Linear regression analysis was adjusted for * sex and *APOE* ε4 carrier status; ^†^ sex; ^‡^ age, sex, disease duration, and *ApoE* ε4 carrier status; ^$^ age, sex, and disease duration.

## Data Availability

The data presented in this study are available on request from the corresponding authors.

## Supplementary Materials

The following supporting information can be downloaded at: https://www.mdpi.com/article/10.3390/life13040882/s1, Table S1: Association between *MMP2* rs243866 (−1575 G/A) SNP and LOAD in cohorts stratified according to the *APOE* ε4 carrier status; Table S2: Association between *MMP2* rs2285053 (−735 C/T) SNP and LOAD in cohorts stratified according to the *APOE* ε4 carrier status.

## References

[1] McKhann G., Drachman D., Folstein M., Katzman R., Price D., Stadlan E.M. (1984). Clinical diagnosis of Alzheimer’s disease: Report of the NINCDS-ADRDA Work Group under the auspices of Department of Health and Human Services Task Force on Alzheimer’s Disease. Neurology.

[2] Niu H., Álvarez-Álvarez I., Guillén-Grima F., Aguinaga-Ontoso I. (2017). Prevalence and incidence of Alzheimer’s disease in Europe: A meta-analysis. Neurologia.

[3] Goedert M., Spillantini M.G., Cairns N.J., Crowther R.A. (1992). Tau proteins of Alzheimer paired helical filaments: Abnormal phosphorylation of all six brain isoforms. Neuron.

[4] Sergeant N., Bretteville A., Hamdane M., Caillet-Boudin M.L., Grognet P., Bombois S., Blum D., Delacourte A., Pasquier F., Vanmechelen E. (2008). Biochemistry of Tau in Alzheimer’s disease and related neurological disorders. Expert. Rev. Proteom..

[5] Behl T., Kaur G., Sehgal A., Bhardwaj S., Singh S., Buhas C., Judea-Pusta C., Uivarosan D., Munteanu M.A., Bungau S. (2021). Multifaceted Role of Matrix Metalloproteinases in Neurodegenerative Diseases: Pathophysiological and Therapeutic Perspectives. Int. J. Mol. Sci..

[6] Singh D., Srivastava S.K., Chaudhuri T.K., Upadhyay G. (2015). Multifaceted role of matrix metalloproteinases (MMPs). Front. Mol. Biosci..

[7] Apte S.S., Parks W.C. (2015). Metalloproteinases: A parade of functions in matrix biology and an outlook for the future. Matrix Biol..

[8] Rivera S., García-González L., Khrestchatisky M., Baranger K. (2019). Metalloproteinases and their tissue inhibitors in Alzheimer’s disease and other neurodegenerative disorders. Cell. Mol. Life Sci..

[9] Rempe R.G., Hartz A.M.S., Bauer B. (2016). Matrix metalloproteinases in the brain and blood-brain barrier: Versatile breakers and makers. J. Cereb. Blood Flow Metab..

[10] Verslegers M., Lemmens K., Van Hove I., Moons L. (2013). Matrix metalloproteinase-2 and -9 as promising benefactors in development, plasticity and repair of the nervous system. Prog. Neurobiol..

[11] Yin K.J., Cirrito J.R., Yan P., Hu X., Xiao Q., Pan X., Bateman R., Song H., Hsu F.F., Turk J. (2006). Matrix metalloproteinases expressed by astrocytes mediate extracellular amyloid-beta peptide catabolism. J. Neurosci..

[12] Lim N.K., Villemagne V.L., Soon C.P., Laughton K.M., Rowe C.C., McLean C.A., Masters C.L., Evin G., Li Q.X. (2011). Investigation of matrix metalloproteinases, MMP-2 and MMP-9, in plasma reveals a decrease of MMP-2 in Alzheimer’s disease. J. Alzheimer’s Dis..

[13] Du H., Li P., Wang J., Qing X., Li W. (2012). The interaction of amyloid β and the receptor for advanced glycation endproducts induces matrix metalloproteinase-2 expression in brain endothelial cells. Cell. Mol. Neurobiol..

[14] Tuna G., Yener G.G., Oktay G., İşlekel G.H., Kİrkalİ F.G. (2018). Evaluation of Matrix Metalloproteinase-2 (MMP-2) and -9 (MMP-9) and Their Tissue Inhibitors (TIMP-1 and TIMP-2) in Plasma from Patients with Neurodegenerative Dementia. J. Alzheimer’s Dis..

[15] Py N.A., Bonnet A.E., Bernard A., Marchalant Y., Charrat E., Checler F., Khrestchatisky M., Baranger K., Rivera S. (2014). Differential spatio-temporal regulation of MMPs in the 5xFAD mouse model of Alzheimer’s disease: Evidence for a pro-amyloidogenic role of MT1-MMP. Front. Aging Neurosci..

[16] Terni B., Ferrer I. (2015). Abnormal Expression and Distribution of MMP2 at Initial Stages of Alzheimer’s Disease-Related Pathology. J. Alzheimer’s Dis..

[17] White A.R., Du T., Laughton K.M., Volitakis I., Sharples R.A., Xilinas M.E., Hoke D.E., Holsinger R.M., Evin G., Cherny R.A. (2006). Degradation of the Alzheimer disease amyloid beta-peptide by metal-dependent up-regulation of metalloprotease activity. J. Biol. Chem..

[18] Li W., Poteet E., Xie L., Liu R., Wen Y., Yang S.H. (2011). Regulation of matrix metalloproteinase 2 by oligomeric amyloid β protein. Brain Res..

[19] Hernandez-Guillamon M., Mawhirt S., Blais S., Montaner J., Neubert T.A., Rostagno A., Ghiso J. (2015). Sequential Amyloid-β Degradation by the Matrix Metalloproteases MMP-2 and MMP-9. J. Biol. Chem..

[20] Mlekusch R., Humpel C. (2009). Matrix metalloproteinases-2 and -3 are reduced in cerebrospinal fluid with low beta-amyloid1-42 levels. Neurosci. Lett..

[21] Brkic M., Balusu S., Van Wonterghem E., Gorlé N., Benilova I., Kremer A., Van Hove I., Moons L., De Strooper B., Kanazir S. (2015). Amyloid β Oligomers Disrupt Blood-CSF Barrier Integrity by Activating Matrix Metalloproteinases. J. Neurosci..

[22] Sweeney M.D., Sagare A.P., Zlokovic B.V. (2018). Blood-brain barrier breakdown in Alzheimer disease and other neurodegenerative disorders. Nat. Rev. Neurol..

[23] Seo J.H., Guo S., Lok J., Navaratna D., Whalen M.J., Kim K.W., Lo E.H. (2012). Neurovascular matrix metalloproteinases and the blood-brain barrier. Curr. Pharm. Des..

[24] Kimura N., Aikawa M., Etou K., Aso Y., Matsubara E. (2020). Association between Matrix Metalloproteinases, Their Tissue Inhibitor and White Matter Lesions in Mild Cognitive Impairment. Curr. Alzheimer Res..

[25] Bjerke M., Zetterberg H., Edman Å., Blennow K., Wallin A., Andreasson U. (2011). Cerebrospinal fluid matrix metalloproteinases and tissue inhibitor of metalloproteinases in combination with subcortical and cortical biomarkers in vascular dementia and Alzheimer’s disease. J. Alzheimer’s Dis..

[26] Khan W., Aguilar C., Kiddle S.J., Doyle O., Thambisetty M., Muehlboeck S., Sattlecker M., Newhouse S., Lovestone S., Dobson R. (2015). A Subset of Cerebrospinal Fluid Proteins from a Multi-Analyte Panel Associated with Brain Atrophy, Disease Classification and Prediction in Alzheimer’s Disease. PLoS ONE.

[27] Harendza S., Lovett D.H., Panzer U., Lukacs Z., Kuhnl P., Stahl R.A. (2003). Linked common polymorphisms in the gelatinase a promoter are associated with diminished transcriptional response to estrogen and genetic fitness. J. Biol. Chem..

[28] Wang F., Jin X.P., Zhu M., Lin X.F., Hu X.F., Wang W.F., Han Z., Huang L.Z. (2011). Genotype association of C(-735)T polymorphism of the MMP-2 gene with the risk of carotid atherosclerosis-vulnerable plaque in the Han Chinese population. Vasc. Med..

[29] Yu C., Zhou Y., Miao X., Xiong P., Tan W., Lin D. (2004). Functional haplotypes in the promoter of matrix metalloproteinase-2 predict risk of the occurrence and metastasis of esophageal cancer. Cancer Res..

[30] Singh H., Marathe S.D., Nema V., Ghate M.V., Gangakhedkar R.R. (2016). Genetic variation of MMP-2(-735 C>T) and MMP-9(-1562 C>T) gene in risk of development of HAND and severity of HAND. J. Gene Med..

[31] Nasreddine Z.S., Phillips N.A., Bédirian V., Charbonneau S., Whitehead V., Collin I., Cummings J.L., Chertkow H. (2005). The Montreal Cognitive Assessment, MoCA: A brief screening tool for mild cognitive impairment. J. Am. Geriatr. Soc..

[32] Miller S.A., Dykes D.D., Polesky H.F. (1988). A simple salting out procedure for extracting DNA from human nucleated cells. Nucleic Acids Res..

[33] Durmanova V., Parnicka Z., Javor J., Minarik G., Vrazda L., Vaseckova B., Gmitterova K., Kralova M., Pecenak J., Filipcik P. (2018). A novel association of polymorphism in the ITGA4 gene encoding the VLA-4 α4 subunit with increased risk of Alzheimer’s disease. Mediat. Inflamm..

[34] Gannoun M.B.A., Raguema N., Zitouni H., Mehdi M., Seda O., Mahjoub T., Lavoie J.L. (2021). MMP-2 and MMP-9 Polymorphisms and Preeclampsia Risk in Tunisian Arabs: A Case-Control Study. J. Clin. Med..

[35] Kiani A., Kamankesh M., Vaisi-Raygani A., Moradi M.R., Tanhapour M., Rahimi Z., Elahi-Rad S., Bahrehmand F., Aliyari M., Aghaz F. (2020). Activities and polymorphisms of MMP-2 and MMP-9, smoking, diabetes and risk of prostate cancer. Mol. Biol. Rep..

[36] Solé X., Guinó E., Valls J., Iniesta R., Moreno V. (2006). SNPStats: A web tool for the analysis of association studies. Bioinformatics.

[37] Cortina-Borja M., Smith A.D., Combarros O., Lehmann D.J. (2009). The synergy factor: A statistic to measure interactions in complex diseases. BMC Res. Notes.

[38] Rahimi Z., Abdan Z., Rahimi Z., Razazian N., Shiri H., Vaisi-Raygani A., Shakiba E., Vessal M., Moradi M.T. (2016). Functional Promoter Polymorphisms of MMP-2 C-735T and MMP-9 C-1562T and Their Synergism with MMP-7 A-181G in Multiple Sclerosis. Immunol. Investig..

[39] Li Y., Ouyang Q.R., Li J., Chen X.R., Li L.L., Xu L., Yu M. (2021). Correlation between matrix metalloproteinase-2 polymorphisms and first and recurrent atherosclerotic ischemic stroke events: A case-control study. J. Int. Med. Res..

[40] Yari K., Rahimi Z., Moradi M.T., Rahimi Z. (2014). The MMP-2 -735 C allele is a risk factor for susceptibility to breast cancer. Asian Pac. J. Cancer Prev..

[41] Yan Y., Fang L., Li Y., Yu Y., Li Y., Cheng J.C., Sun Y.P. (2021). Association of MMP2 and MMP9 gene polymorphisms with the recurrent spontaneous abortion: A meta-analysis. Gene.

[42] Rahimi Z., Lotfi S., Ahmadi A., Jalilian N., Shakiba E., Vaisi-Raygani A., Rahimi Z. (2018). Matrix metalloproteinase-2 C-735T and its interaction with matrix metalloproteinase-7 A-181G polymorphism are associated with the risk of preeclampsia: Influence on total antioxidant capacity and blood pressure. J. Obstet. Gynaecol..

[43] Peng B., Cao L., Ma X., Wang W., Wang D., Yu L. (2010). Meta-analysis of association between matrix metalloproteinases 2, 7 and 9 promoter polymorphisms and cancer risk. Mutagenesis.

[44] Xu E., Xia X., Lü B., Xing X., Huang Q., Ma Y., Wang W., Lai M. (2007). Association of matrix metalloproteinase-2 and -9 promoter polymorphisms with colorectal cancer in Chinese. Mol. Carcinog..

[45] Tarki S.E., Far I.S., Aminimoghaddam S., Fooladi B., Sarhangi N., Farahani M.S., Klashami Z.N., Hamidi A.K., Amoli M.M. (2021). Investigating the association of matrix metalloproteinase-2 gene variants with endometriosis in an Iranian population. Eur. J. Obstet. Gynecol. Reprod. Biol..

[46] Yadav S.S., Mandal R.K., Singh M.K., Usman K., Khattri S. (2014). Genetic variants of matrix metalloproteinase (MMP2) gene influence metabolic syndrome susceptibility. Genet. Test. Mol. Biomark..

[47] Pérez-Hernández N., Vargas-Alarcón G., Martínez-Rodríguez N., Martínez-Ríos M.A., Peña-Duque M.A., Peña-Díaz Ade L., Valente-Acosta B., Posadas-Romero C., Medina A., Rodríguez-Pérez J.M. (2012). The matrix metalloproteinase 2-1575 gene polymorphism is associated with the risk of developing myocardial infarction in Mexican patients. J. Atheroscler. Thromb..

[48] Corder E.H., Saunders A.M., Strittmatter W.J., Schmechel D.E., Gaskell P.C., Small G.W., Roses A.D., Haines J.L., Pericak-Vance M.A. (1993). Gene dose of apolipoprotein E type 4 allele and the risk of Alzheimer’s disease in late onset families. Science.

[49] Farrer L.A., Cupples L.A., Haines J.L., Hyman B., Kukull W.A., Mayeux R., Myers R.H., Pericak-Vance M.A., Risch N., van Duijn C.M. (1997). Effects of age, sex, and ethnicity on the association between apolipoprotein E genotype and Alzheimer disease. A meta-analysis. APOE and Alzheimer Disease Meta Analysis Consortium. JAMA.

[50] Crean S., Ward A., Mercaldi C.J., Collins J.M., Cook M.N., Baker N.L., Arrighi H.M. (2011). Apolipoprotein E ε4 prevalence in Alzheimer’s disease patients varies across global populations: A systematic literature review and meta-analysis. Dement. Geriatr. Cogn. Disord..

[51] Montagne A., Nation D.A., Sagare A.P., Barisano G., Sweeney M.D., Chakhoyan A., Pachicano M., Joe E., Nelson A.R., D’Orazio L.M. (2020). APOE4 leads to blood-brain barrier dysfunction predicting cognitive decline. Nature.

[52] Castellano J.M., Kim J., Stewart F.R., Jiang H., DeMattos R.B., Patterson B.W., Fagan A.M., Morris J.C., Mawuenyega K.G., Cruchaga C. (2011). Human apoE isoforms differentially regulate brain amyloid-β peptide clearance. Sci. Transl. Med..

[53] Mahoney-Sanchez L., Belaidi A.A., Bush A.I., Ayton S. (2016). The Complex Role of Apolipoprotein E in Alzheimer’s Disease: An Overview and Update. J. Mol. Neurosci..

[54] Choi J., Kwon H., Han P.L. (2021). Hyperoxygenation Treatment Reduces Beta-amyloid Deposition via MeCP2-dependent Upregulation of MMP-2 and MMP-9 in the Hippocampus of Tg-APP/PS1 Mice. Exp. Neurobiol..

[55] Garcia-Alloza M., Prada C., Lattarulo C., Fine S., Borrelli L.A., Betensky R., Greenberg S.M., Frosch M.P., Bacskai B.J. (2009). Matrix metalloproteinase inhibition reduces oxidative stress associated with cerebral amyloid angiopathy in vivo in transgenic mice. J. Neurochem..

[56] Wan W., Cao L., Liu L., Zhang C., Kalionis B., Tai X., Li Y., Xia S. (2015). Aβ(1-42) oligomer-induced leakage in an in vitro blood-brain barrier model is associated with up-regulation of RAGE and metalloproteinases, and down-regulation of tight junction scaffold proteins. J. Neurochem..

[57] Hadass O., Tomlinson B.N., Gooyit M., Chen S., Purdy J.J., Walker J.M., Zhang C., Giritharan A.B., Purnell W., Robinson C.R. (2013). Selective inhibition of matrix metalloproteinase-9 attenuates secondary damage resulting from severe traumatic brain injury. PLoS ONE.

[58] Jiang P., Li C., Xiang Z., Jiao B. (2014). Tanshinone IIA reduces the risk of Alzheimer’s disease by inhibiting iNOS, MMP-2 and NF-κBp65 transcription and translation in the temporal lobes of rat models of Alzheimer’s disease. Mol. Med. Rep..

