# Supplementary material for: MMP2 rs243866 and rs2285053 Polymorphisms and Alzheimer’s Disease Risk in Slovak Caucasian Population
Source: Life (Basel). 2023 Mar 26;13(4):882. doi: 10.3390/life13040882 (PMC10143987; doi:10.3390/life13040882)
Supplement: Supplementary file 1 [file life-13-00882-s001.zip › life-2209670-supplementary.pdf]

Supplementary materials

Table S1. Association between *MMP2* rs243866 (-1575 G/A) SNP and LOAD in cohorts stratified according to the *APOE ε4* carrier status.

Allele / Genotype	LOAD	Controls	Genetic model	Logistic regression analysis *p*-Value	OR (95% CI)
***APOE ε4*-positive cohort**					
			Allele contrast (A vs. G)	0.70	1.11 (0.66–1.85)
G	135 (74.18%)	105 (76.09%)	Codominant (GA vs. GG)	0.40	0.72 (0.35–1.51)
A	47 (25.82%)	33 (23.91%)	Codominant (AA vs. GG)	0.58	1.46 (0.45–4.79)
GG	55 (60.44%)	41 (59.42%)	Dominant (AA + GA vs. GG)	0.65	0.86 (0.44–1.68)
GA	25 (27.47%)	23 (33.33%)	Recessive (AA vs. GA + GG)	0.40	1.63 (0.51–5.19)
AA	11 (12.09%)	5 (7.25%)	Over-dominant (GA vs. GG + AA)	0.31	0.69 (0.34–1.41)
			Log-additive	0.97	1.01 (0.62–1.65)
***APOE ε4*-negative cohort**					
			Allele contrast (A vs. G)	0.53	1.11 (0.80–1.56)
G	180 (72.58%)	454 (74.67%)	Codominant (GA vs. GG)	0.91	0.98 (0.62–1.54)
A	68 (27.42%)	154 (25.33%)	Codominant (AA vs. GG)	0.30	1.51 (0.69–3.29)
GG	68 (54.84%)	171 (56.25%)	Dominant (AA + GA vs. GG)	0.80	1.06 (0.69–1.62)
GA	44 (35.48%)	112 (36.84%)	Recessive (AA vs. GA + GG)	0.29	1.52 (0.71–3.26)
AA	12 (9.68%)	21 (6.91%)	Over-dominant (GA vs. GG + AA)	0.74	0.93 (0.59–1.45)
			Log-additive	0.52	1.12 (0.80–1.56)

Allele and genotype counts are presented with frequencies in parentheses. *p*, OR, and 95% CI values for genotype comparisons were adjusted for age and sex. CI: confidence interval; LOAD: late-onset Alzheimer's disease; OR: odds ratio.

Table S2. Association between *MMP2* rs2285053 (-735 C/T) SNP and LOAD in cohorts stratified according to the *APOE ε4* carrier status.

Allele / Genotype	LOAD	Controls	Genetic model	Logistic regression analysis *p*-Value	OR (95% CI)
***APOE ε4*-positive cohort**					
			Allele contrast (T vs. C)	0.93	1.03 (0.55–1.92)
C	155 (85.16%)	118 (85.51%)	Codominant (CT vs. CC)	0.57	1.24 (0.57–2.72)
T	27 (14.84%)	20 (14.49%)	Codominant (TT vs. CC)	0.37	0.34 (0.03–3.72)
CC	65 (71.14%)	52 (75.36%)	Dominant (TT + CT vs. CC)	0.79	1.11 (0.52–2.34)
CT	25 (27.47%)	14 (20.29%)	Recessive (TT vs. CT + CC)	0.33	0.32 (0.03–3.52)
TT	1 (1.09%)	3 (4.35%)	Over-dominant (CT vs. CC + TT)	0.53	1.28 (0.59–2.80)
			Log-additive	0.95	0.98 (0.51–1.87)
***APOE ε4*-negative cohort**					
			Allele contrast (T vs. C)	0.39	1.21 (0.79–1.85)
C	212 (85.48%)	533 (87.66%)	Codominant (CT vs. CC)	0.40	1.23 (0.76–1.98)
T	36 (14.52%)	75 (12.34%)	Codominant (TT vs. CC)	0.53	0.87 (0.04–21.53)
CC	88 (70.97%)	230 (75.66%)	Dominant (TT + CT vs. CC)	0.43	1.22 (0.75–1.96)
CT	36 (29.03%)	73 (24.01%)	Recessive (TT vs. CT + CC)	0.52	0.81 (0.03–20.10)
TT	0 (0.00%)	1 (0.33%)	Over-dominant (CT vs. CC + TT)	0.40	1.23 (0.76–1.99)
			Log-additive	0.46	1.20 (0.75–1.92)

Allele and genotype counts are presented with frequencies in parentheses. *p*, OR, and 95% CI values for genotype comparisons were adjusted for age and sex. CI: confidence interval; LOAD: late-onset Alzheimer's disease; OR: odds ratio.
